# Determination of the Effects of Pear-*Morchella* Intercropping Mode on *M. sextelata* Quality, Yield, and Soil Microbial Community

**DOI:** 10.3390/jof10110759

**Published:** 2024-11-01

**Authors:** Xiao Liu, Jun-Zhe Liu, Jing Liu, Jing Zhang, Chun-Lei Wang

**Affiliations:** College of Horticulture and Landscape Architecture, Yangzhou University, Yangzhou 225009, China; liuxiao@yzu.edu.cn (X.L.); 17851971612@163.com (J.-Z.L.); liujing1026044@163.com (J.L.); zhangj45@yzu.edu.cn (J.Z.)

**Keywords:** intercropping, *Morchella*, yield, nutrient bag, microbial community

## Abstract

The intercropping of *Morchella* in pear orchards has important production value in improving the utilization rate and economic benefits of the orchard; however, there is little research on the intercropping model of pear-*Morchella*. In this study, metabolomics analysis found that compared with greenhouse cultivation, there were 104 and 142 metabolites significantly increased and decreased in the intercropping mode of *M. sextelata*, respectively. Among them, there was a significant accumulation of amino acids (phenylalanine, lysine, proline, citrulline, and ornithine), sugars (arabinitol and glucosamine), and organic acids (quinic acid, fumaric acid, and malic acid) related to the unique taste of *Morchella* in intercropping cultivation. In addition, research on the cultivation model using exogenous nutrient bags indicated that placing the density of six exogenous nutrient bags per square meter was most suitable for yield formation. Adding pear sawdust to the nutrient bags (PN) significantly increased the yield of morel per unit area. Moreover, soil microbial community analysis showed that fungal alpha diversity dramatically declined in PN-cultivated soil, which decreased the relative abundance of soil-borne fungal pathogens, including *Fusarium* and *Aspergillus*. Some beneficial soil bacteria abundance increased in the PN-used soil, such as *Pedobacter*, *Pseudomonas,* and *Devosia*. This study provides novel insights into the effects of intercropping on the internal quality of *Morchella* and enriches the theoretical knowledge on the consummation of the pear-*Morchella* model formation, further improving agricultural resource utilization efficiency and crop productivity.

## 1. Introduction

Intercropping has been widely used in fruit production because it can maximize crop productivity and economic benefits. Fruit-crop intercropping models have been relatively well developed, such as banana intercropped with pumpkin [1], strawberry intercropped with broad bean [2], or cherry intercropped with *Solanum photeinocarpum* [3]. However, with the increase in tree age, most trees in fruit orchards lack light between rows; thus, intercropping conventional crops will suffer from poor growth and low yield, and it leads some grain and cash crops to be withdrawn from the intercropping production mode. In addition, agricultural planting and production costs, including agricultural material prices, labor costs, and land lease prices, have continued to grow in recent years. Based on this, the intercropping of cost-effective cash crops is imperative. Pear is one of the important fruit industries and has a wide planting area in China. Moreover, pear belongs to the Rosaceae deciduous tree species, and its production has a long winter dormancy period. Thus, how to use the intercropping space of pear orchards to fully tap the planting potential is an important research direction for scientific researchers.

*Morchella* is a prized edible and nutritional fungus, of which the fruiting body is rich in polysaccharides, amino acids, vitamins, and other nutrients with anti-cancer, anti-fatigue, and immunoregulatory activities [4,5,6]. The intercropping of *Morchella* in the fruit orchard has many advantages, such as making full use of the light, heat, and land resources in the fruit orchard to inhibit the growth of weeds, improve the ecological environment and microclimate of the orchard, and prevent the occurrence of diseases and pests. In addition, this intercropping model also effectively increases the economic income of farmers. *Morchella* intercropping studies have been reported. For instance, a previous study found that peach-*Morchella* intercropping improves soil structure and fertility while decreasing soil fungal diversity, which can contribute to greater economic benefits [7]. To date, there are few reports on the intercropping of *Morchella* in pear orchards. At present, the production of *Morchella* is mainly carried out in the form of facility cultivation [8]. Although this cultivation form has a higher yield, the input cost is also high. Further analysis is needed to determine the differences in the quality of *Morchella* cultivated through simple facilities in pear orchards compared to others.

Researchers have extensively explored the repeatability of the cultivation of *Morchella* in different cultivation environments, such as appropriate bacterial strain status, fertilization, exogenous growth regulators, physical treatment of soil, or nutrient bag improvements [9,10,11,12,13]. The technology of adding exogenous nutrient bags is the key to ensuring efficient commercial production of *Morchella*. Further research is needed to determine the appropriate nutrient bag density for intercropping *Morchella* in pear orchards. In addition, in a study of *Pleurotus ostreatus* mushrooms, it was found that pear sawdust is a very good cultivation substrate [14]. Thus, further research is needed to determine the effect of using pear sawdust as an exogenous nutrient bag component on the growth of *Morchella*. *M. sextelata* has good economic value because of its thick meat head, dark color, and high price, and therefore it is the main cultivated variety of *Morchella* in China [15]. This study aims to provide a theoretical basis for improving the quality and efficiency of pear orchards and formulating the technical specifications for intercropping *M. sextelata* in pear orchards.

## 2. Materials and Methods

### 2.1. Experiment Design

To compare the changes in the intrinsic quality of *M. sextelata* under the intercropping mode, we cultivated them separately in pear orchards and greenhouses. The *M. sextelata* samples were sown in the pear orchard of Yangzhou University in November 2021. One month before sowing, the soil was mixed with about 80 g/m^2^ of quick lime to sterilize the soil and prevent the occurrence of pests and diseases in the soil in the later stage. The spawn and exogenous nutrient bags were provided by Sichuan Junzhiwei Agricultural Development Co., Ltd. Chengdu, China. The exogenous nutrient bag provides nutritional support for the growth of morel mycelium in the soil. *Morchella* strain was sown on the ridge along with the soil. Subsequently, the ridge was covered with polyethylene transparent film and shade net (shading rate of 70%) for moisturizing and shading, respectively. About ten days later, the hypha started to grow out of the soil; then, the nutrient bags were placed in the field with a density of five bags per m^2^. The nutrient bags’ main ingredients were wheat and rice husk at a ratio of approximately 65%:35%, and an additional 5 g of quick lime was added, with each bag containing approximately 500–600 g. Mix the ingredients in the nutrient bag in proportion and perform high-temperature and high-pressure sterilization. Then, *Morchella* fruiting bodies gradually formed. When the color and shape of the fungal cap no longer change, it is considered mature and harvested in batches. Next year, the full mature fruiting bodies were sampled on March 26. The timeline of *M. sextelata* cultivated in the greenhouse was the same as in the pear orchard. Thus, the collected *M. sextelata* samples were divided into orchard cultivation (OC) or protection cultivation (PC).

In addition, nutrient bags are crucial for the growth of morel mushrooms. Therefore, we have implemented the following two treatments for the cultivation and improvement of nutrient bags.

To compare the effects of different nutrient bag densities on the yield of *Morchella*. In November 2023, the *M. sextelata* samples were sown in the pear orchard of Yangzhou University. This experiment compared the densities of 4, 6, and 8 nutrient bags per m^2^, respectively, on the impact of *M. sextelata* biomass and yield. Each treatment involved planting four square meters. When the *Morchella* fruiting bodies matured, the single weight, longitudinal, and transverse lengths of each fruiting body were calculated separately, and the distribution ratio of single fruit weight was calculated. The average yield is obtained by dividing the total weight of each processed fruiting body by the cultivation area.

At present, pear sawdust is used as a mushroom culture medium in production, which not only effectively utilizes the waste of pear trees but also plays an important role in increasing yield and income for farmers. To investigate whether adding pear sawdust to nutrient bags would affect the yield of *Morchella*, in the current experiment, the formula ratio of the reference nutrient bag was wheat grains 48%, corn cob 30%, cottonseed husk 10%, wheat bran 10%, lime 1%, and gypsum 1%, respectively (N). In addition, replace half (H) or all (A) of the corn cobs with pear sawdust in other nutrient bag formula modifications. Each bag weighs approximately 700–800 g. Mix the ingredients in the nutrient bag in proportion and perform high-temperature and high-pressure sterilization. The planting density is four bags per square meter for each treatment. The other cultivation modes and statistical methods are the same as above in 2023.

### 2.2. Metabolomics Analysis

Three samples of fully mature *M. sextelata* fruiting bodies from OC or PC were used in the LC-MS/MS system for analysis. UHPLC-MS/MS analyses were performed using a Vanquish UHPLC system (Thermo Fisher, Waltham, MA, USA) coupled with an Orbitrap Q Exactive^TM^ HF mass spectrometer (Thermo Fisher, Bremen, Germany) in Novogene Co., Ltd. (Beijing, China). The raw data files generated by UHPLC-MS/MS were processed using Compound Discoverer 3.1 (CD3.1, Thermo Fisher, Hercules, CA, USA) to perform peak alignment, peak picking, and quantitation for each metabolite. The metabolites with a VIP > 1, a *p*-value < 0.05, and a fold change ≥ 1.5 or ≤0.667 were considered to be differential metabolites (DEMs).

### 2.3. Soil Microbial Analysis

Soils from all plots were collected after intercropping with normal nutrient bags (NN) or nutrient bags improved by adding pear sawdust (PN) in 2023. Each field was collected (0–20 cm depth), and the six-point sampling method was used to collect soil samples, and each point weighed approximately 500 g. Then, six samples were mixed from each field, and three samples of equal quality were randomly selected for subsequent DNA extraction.

The V3–V4 hypervariable regions of bacterial 16S rRNA were amplified using barcoded primers 338F and 806R, and fungal ITS1 regions were targeted for amplification through two rounds of PCR. The purified PCR amplicons were sequenced using the Illumina NovaSeq platform from Biomarker Technology Co., Ltd. (Beijing, China). The bioinformatics analysis of this study was performed with the aid of the BMKCloud (http://www.biocloud.net/ on 22 April 2024). The Alpha diversity was calculated and displayed by the QIIME2 (versoin 2020.6) and R software, respectively. Furthermore, we employed Linear Discriminant Analysis (LDA) effect size (LEfSe) to test the significant taxonomic difference among groups. A logarithmic LDA score of 4.0 was set as the threshold for discriminative features. To explore the dissimilarities of the microbiome among different factors, a redundancy analysis (RDA) was performed in R using the package ‘vegan’.

### 2.4. Statistical Analysis

The data were analyzed using variance analysis (ANOVA) and *t*-test by GraphPad Prism7.0. Differences were considered significant at *p* < 0.05. Bar graphs were drawn using GraphPad Prism.

## 3. Results

### 3.1. Metabolomics Analysis of M. sextelata Intercropped in Pear Orchard

**Figure 1:**
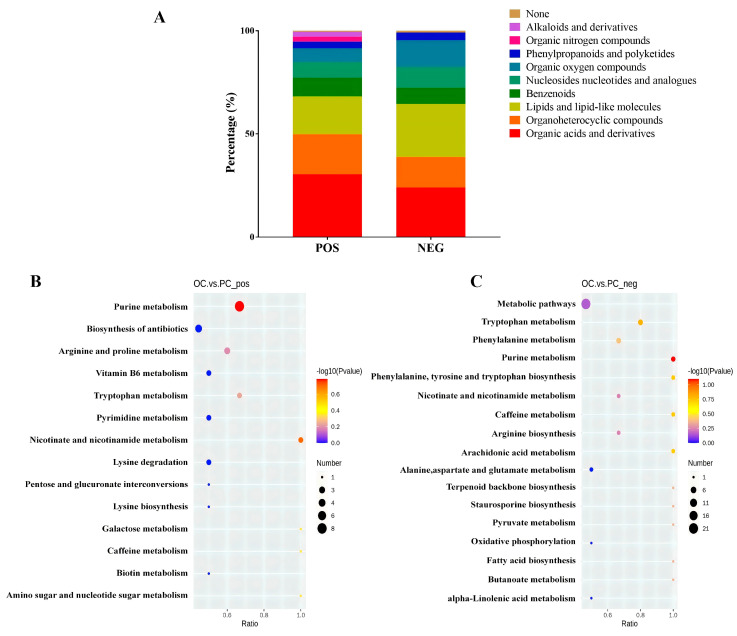
Metabolomics analysis of *M. sextelata* intercropped in pear orchard. (**A**) refers to the annotated metabolites classification. (**B**,**C**) refer to KEGG analysis in *M. sextelata* under the POS or NEG model, respectively. OC represents orchard cultivation; PC represents protection cultivation.

A total of 313 annotated metabolites were identified in *M. sextelata* under the POS model, of which 210 were classified into 9 groups. Among them, organic acids and derivatives, organoheterocyclic compounds, lipids, and lipid-like molecules were the main enriched categories (Figure 1A). In addition, 244 annotated metabolites were identified under the NEG model and 216 were classified into 7 groups. In detail, lipids and lipid-like molecules, organic acids and derivatives, organoheterocyclic compounds, organooxygen compounds, nucleosides, nucleotides, and analogs were the main enriched category (Figure 1A).

A comparative analysis was performed to identify differences in the metabolite expression patterns. A VIP > 1, *p*-value < 0.05, and fold change ≥ 1.5 or ≤0.667 were considered as screening criteria for significant differences. The PLS-DA results showed that 140 (66 up-regulated and 74 down-regulated) and 106 (38 up-regulated and 68 down-regulated) were identified in the POS model and the NEG model, respectively. Kyoto Encyclopedia of Genes and Genomes (KEGG) enrichment analysis showed the DEMs were enriched in pathways involving purine metabolism, nicotinate and nicotinamide metabolism, galactose metabolism, caffeine metabolism, amino sugar and nucleotide sugar metabolism, tryptophan metabolism, and arginine and proline metabolism under the POS model (Figure 1B). Moreover, the pathways of purine metabolism, tryptophan metabolism, caffeine metabolism, phenylalanine, tyrosine and tryptophan biosynthesis, arachidonic acid metabolism, phenylalanine metabolism, and fatty acid biosynthesis were mainly enriched under the NEG model (Figure 1C).

### 3.2. Analysis of Differential Amino Acids, Sugars, and Acids of Intercropped M. sextelata

As shown in Table 1, the content of phenylalanine, lysine, proline, citrulline, ornithine, and tryptophan significantly changed in the *M. sextelata* intercropped in pear orchard. In detail, phenylalanine, lysine, proline, citrulline, and ornithine were significantly accumulated, while tryptophan significantly decreased. Moreover, arabinitol, glucosamine, quinic acid, fumaric acid, and malic acid content in intercropped *M. sextelata* were significantly increased, while mannose content was significantly decreased.

### 3.3. The Effect of Different Nutrient Bag Densities on the Biomass and Yield of Morchella

**Figure 2:**
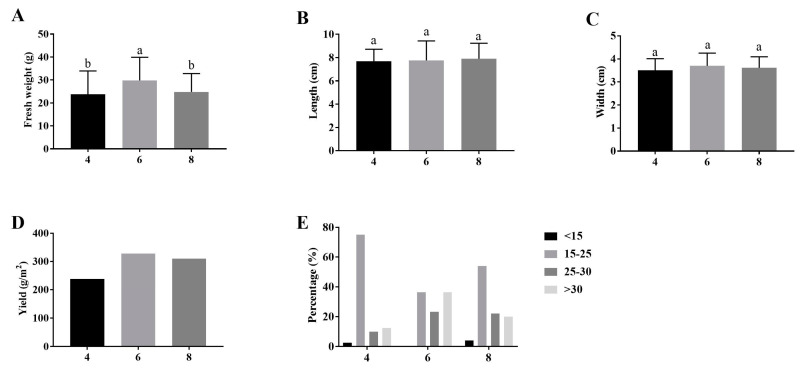
The effect of different nutrient bag densities on the biomass and yield of *M. sextelata*. (**A**) Single fruiting body fresh weight. (**B**) Fruiting body length. (**C**) Fruiting body width. (**D**) Yield of per m^2^. (**E**) Fruiting body fresh weight distribution. Different letters indicate significant differences at *p* < 0.05.

*Morchella* biomass, fruiting body yield, and harvest index resulting from the comparison of the nutrient bag densities are shown in Figure 2. Under six nutrient bag densities, *Morchella* had higher single fresh weight than the other nutrient bag densities (Figure 2A). No significant difference in fruiting body length and width was observed in each treatment (Figure 2B,C). As shown in Figure 2D, the yield of four, six, and eight nutrient bag densities treatments was 237.73 g/m^2^, 327.81 g/m^2^, and 310.15 g/m^2^, respectively (Figure 2D). Moreover, the single fruiting body fresh weight of *Morchella* under four or eight nutrient bag densities was predominately distributed in 15 g~25 g. Under the six nutrient bag densities cultivation, the single weight of *Morchella* was higher than 15 g, and the distribution within each range was relatively uniform (Figure 2E).

### 3.4. The Effect of Different Nutrient Bag Formulae on the Biomass and Yield of Morchella

**Figure 3:**
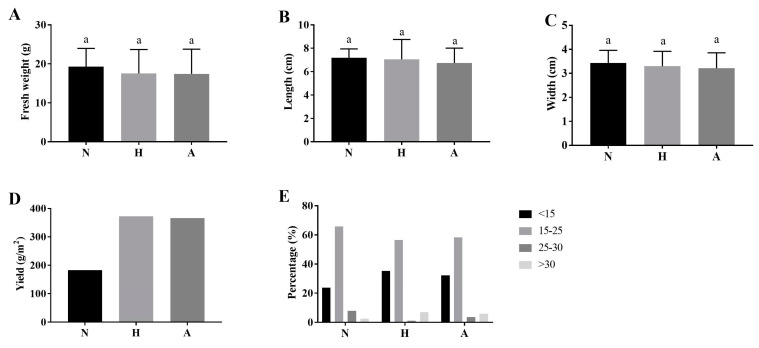
The effect of different nutrient bag formulae on the biomass and yield of *M. sextelata*. (**A**) Single fruiting body fresh weight. (**B**) Fruiting body length. (**C**) Fruiting body width. (**D**) Yield of per m^2^. (**E**) Fruiting body fresh weight distribution. N, H, and A refer to the proportions of pear sawdust in the nutrient bag: none, half, and all, respectively. Different letters indicate significant differences at *p* < 0.05.

In order to test the effect of pear sawdust on the growth and yield of *Morchella*, we added pear sawdust to nutrient bags in different proportions and used it for the cultivation of *Morchella*. As shown in Figure 3A–C, the fruiting body had no significant difference in single fresh weight, length, and width in each pear sawdust proportion treatment. Under the cultivation of nutrient bags mixed with half the proportion of pear sawdust (H) or the whole proportion of sawdust (A), the yield of *Morchella* was 372.91 g/m^2^ and 366.55 g/m^2^, respectively, which was significantly higher than that of the control group (N) (182.89 g/m^2^) (Figure 3D). Moreover, the single fruiting body fresh weight of *Morchella* under each treatment was predominately distributed in 15 g~25 g. However, the H and A fresh weight proportions of *Morchella* below 15 g also had a high level (Figure 3E).

### 3.5. Composition and Diversity of the Soil Bacterial Community from Different Nutrient Bag Cultivation Methods

**Figure 4:**
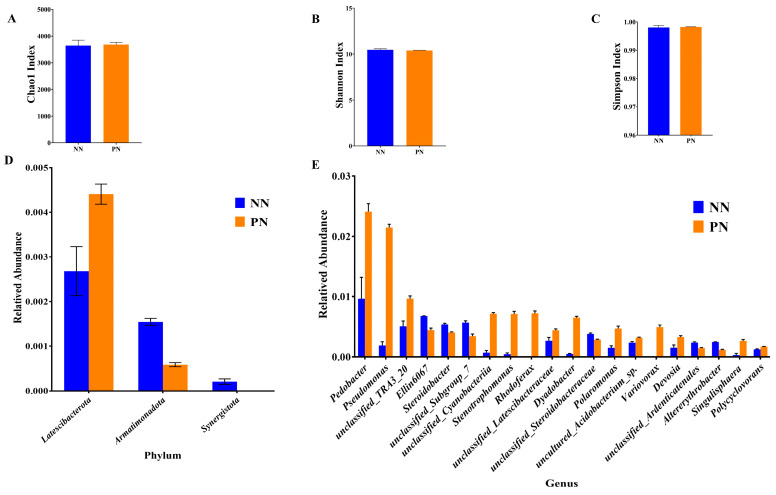
Composition and diversity of the soil bacterial community cultivated in different nutrient bags. (**A**) Chao1 index. (**B**) Shannon index. (**C**) Simpson index. (**D**) Different soil bacteria at the phylum level. (**E**) Different soil bacteria at the genus level. NN represents normal nutrient bags; PN represents nutrient bags with added pear sawdust.

We sequenced the V3–V4 region of the 16S rRNA gene and obtained the sequences from the soil samples after intercropping *Morchella*. Rarefaction curves implied that the sequencing coverage was sufficient, as plateaus were reached for soil communities. Based on the total bacterial alpha diversity analysis, the Chao1 index, Shannon index, and Simpson index had no significant difference between PN and NN (Figure 4A–C).

As shown in Figure 4D, the soil from PN had three phylum differences with NN: *Latescibacterota* relative abundance was significantly increased, while *Armatimonadota* and *Synergistota* were significantly decreased, respectively. Moreover, the top 20 genera with the lowest *p*-value were identified. Among them, the relative abundance of *Altererythrobacter*, *Devosia*, *Dyadobacter*, *Pedobacter*, *Polaromonas*, *Polycyclovorans*, *Pseudomonas*, *Rhodoferax*, *Singulisphaera*, *Stenotrophomonas*, unclassified_*Cyanobacteriia*, unclassified_*Latescibacteraceae*, unclassified_*TRA3_20*, uncultured_*Acidobacterium*_sp, and *Variovorax* in PN was significantly higher than that in NN, while the relative abundance of *Ellin6067*, *Steroidobacter*, unclassified_*Ardenticatenales*, unclassified_*Steroidobacteraceae,* and unclassified_*Subgroup_7* was significantly decreased (Figure 4E).

### 3.6. Composition and Diversity of the Soil Fungal Community from Different Nutrient Bag Cultivation Methods

**Figure 5:**
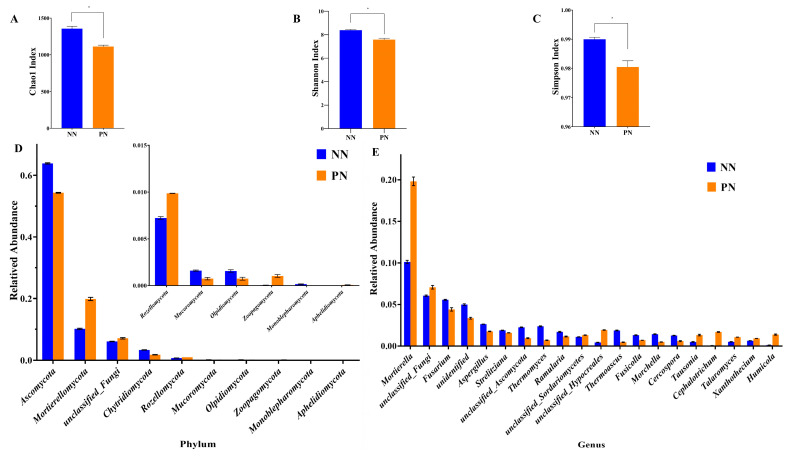
Composition and diversity of the soil fungal community cultivated in different nutrient bags. (**A**) Chao1 index. (**B**) Shannon index. (**C**) Simpson index. (**D**) Different soil fungi at the phylum level. (**E**) Different soil fungi at the genus level. NN represents normal nutrient bags; PN represents nutrient bags with added pear sawdust. The asterisk on the line connecting the columns represents significant differences at *p*-value < 0.05.

Based on the total fungal community estimation, the Chao1 index, Shannon index, and Simpson index were significantly reduced in PN (Figure 5A–C). In terms of phylum, the relative abundance of *Aphelidiomycota*, *Mortierellomycota*, *Rozellomycota,* and *Zoopagomycota* in PN was significantly higher than that in NN (Figure 5D). The top 20 genera with the lowest *p*-value showed that nine fungal genus (*Cephalotrichum, Humicola, Mortierella, Talaromyces, Tausonia*, unclassified_*Hypocreales*, unclassified_*Sordariomycetes*, unclassified_Fungi, *Xanthothecium*) had relative abundances in PN that were significantly higher than those in NN (Figure 5E).

## 4. Discussion

Pear is one of the most important fruit trees in China. However, pear orchards have a long unproductive period after harvest. How to improve the utilization rate of the orchards and improve the economic benefits for farmers is very urgent. *Morchella* is a commercially important edible mushroom with economic and scientific value. In recent years, the domestication and cultivation of *M. esculenta* in China have made great progress and gradually embarked on the road of commercialization [16]. To date, the research on the introduction and cultivation of *Morchella* in central and eastern China has gradually increased, and the exploration of intercropping modes with fruit orchards has also gradually deepened. However, there are few studies on the intercropping of pear orchards and *Morchella*. The components of soluble sugars, organic acids, and free amino acids are the prominent source of the complicated taste of *Morchella*. Glucose, fructose, and galactose are the major soluble monosaccharides in *Morchella* [17]. The polyols in morels are much more abundant than the monosaccharides, which impart the natural sweetness of morels. For instance, mannitol is the predominant sugar alcohol in *M. importuna* fruiting bodies [18]. In addition, succinic acid, fumaric acid, citric acid, and malic acid are the major organic acids in the fruiting body of *M. importuna*. They are also responsible for part of the unique taste of morels [18]. In the current study, we found that compared with greenhouse cultivation, the content of several key amino acids, sugar alcohols, and organic acids in *Morchella* under intercropping cultivation mode showed a significant increase. Although there are certain protective measures in the growth process of morel mushrooms in pear orchards, they are also susceptible to a certain degree of low temperature or soil moisture stress. The significant increase in proline content also confirms this result. Wild morel mushrooms are mainly distributed in colder climates, such as Sichuan and Guizhou in China, with high quality but low yield. Indoor artificial cultivation of morel mushrooms provides suitable environmental conditions, resulting in a significant increase in yield. However, during the process of intercropping in pear orchards, although there were simple facilities to assist the growth of *Morchella*, it was still affected by low temperatures or precipitation. A certain degree of abiotic stress in production is conducive to the formation of intrinsic quality. Therefore, we speculate that the quality improvement of morel mushrooms under the intercropping mode may be related to this.

To increase the yield of *Morchella*, the industry of artificial cultivation of *Morchella* has been rapidly increasing in recent decades. In particular, providing exogenous nutrient bags has made a breakthrough in the development of indoor cultivation [19]. The morel mycelia colonize the soil after spawning under suitable temperatures and humidity. After 10–15 days, a vast expanse of whiteness appears on the surface of the mushroom bed. Then, an exogenous nutrient bag can be placed in the mushroom bed [20]. The substrates used for the exogenous nutrient bag include wheat, chaff, sawdust, and cottonseed hulls. Currently, there is an increasing amount of research on the impact of nutrient bags on the yield and quality of morel mushrooms. Exogenous nutrient bag decomposition leads to a rapid increase in the organic carbon content in the surface soil of the mushroom bed, which was thereafter consumed during morel fruiting [21,22]. In this study, we found that the single weight and yield of *Morchella* under six nutrient bag density cultivation were the highest, and the proportion of high single fruit weight was also relatively high. Therefore, this density may be more suitable for *Morchella* intercropping in pear orchards. Moreover, pear trees produce a large amount of discarded branches after pruning in winter. Pear branches are rich in nutrients, and there have been studies that have turned pear branches into sawdust for the production of edible mushrooms and achieved good results. Terashima et al. used sand pear sawdust as a substrate for *Pleurotus ostreatus* cultivation and found that fruit body yield on the pear sawdust substrate was as high as that on Sugi sawdust substrate, which is regularly used in Pleurotus cultivation [14]. Chemical analysis showed that the pear sawdust contained more free sugars needed for mycelial growth than Sugi sawdust. In this study, we found that adding pear sawdust nutrient bags can significantly increase the yield of *Morchella* per unit area. This indicates that incorporating pear sawdust into the production of *Morchella* and promoting the formation of a pear-*Morchella* cycle cultivation model is highly feasible, but further research is needed to optimize the proportion of nutrient bag formulations.

Soil bacteria, fungi, or archaea play a significant role in conserving multiple soil ecosystem functions, including soil nutrient cycling and soil organic matter content and composition. Previous studies found that soil moisture and available K and P accumulation by *M. crassipes* were affected in inoculated plants and resulted in growth enhancements that were equal to those of the plants treated with urea. The inoculation with *M. crassipes* had a positive effect on maize yield, making the crop system more sustainable. *M. crassipes* has the potential to become a supplement or an alternative to urea fertilizers [23]. Peach-*Morchella* intercropping mode obviously enhanced soil enzyme activities and mineral absorption and transformation in peach orchard soils. The intercropping also resulted in a decline in soil fungal diversity, and the 2-year soil samples showed a higher abundance of *Zygomycota* [7]. A previous study also found that the *Morchella*-cultivated soil significantly affects the potassium content of the soil, which can directly or indirectly promote *Morchella* yield by inhibiting soil fungal richness [24]. The results showed that during the first year, *M. sextelata* mycelium overwhelmed the resident soil fungal community by reducing the alpha diversity and niche breadth of soil fungal patterns by a greater amount compared to the continuous cropping regime, leading to high crop yield of *M. sextelata* [25]. Dazomet is a broad-spectrum soil fumigant that is used to control soil pests, weeds, and pathogens by releasing methylisothiocyanate, formaldehyde, monomethylamine, and hydrogen sulfide. Previous studies also showed that the dazomet treatment increased *Morchella* yield, which may be closely related to the decrease in the abundance of *Paecilomyces*, *Trichoderma*, *Fusarium*, *Penicillium,* and *Acremonium* or to the increase in the relative abundance of beneficial soil bacteria, including *Bacillius* and *Pseudomonas* [26]. In particular, the role of *Pseudomonas* in the cultivation of button mushrooms and *Morchella* has been shown to increase both yield and primordia formation [8,27,28]. In our study, alpha diversity analysis results showed that using pear sawdust altered the microbial communities in pear orchard soil, which decreased the relative abundance of soil-borne fungal pathogens, including *Fusarium* and *Aspergillus*, and increased the beneficial soil bacteria abundance, including *Pedobacter*, *Pseudomonas,* and *Devosia*. In addition, using pear sawdust as a nutrient bag resulted in a lower abundance of *Morchella* in the soil, and there is a negative correlation with yield. The above results indicate that using pear sawdust as a nutrient bag for cultivation increases the proportion of beneficial microorganisms in the soil, promotes the transformation of *Morchella* mycelium into fruiting bodies, and increases the yield of *Morchella*.

## 5. Conclusions

The intercropping of *Morchella* in pear orchards has important production value in improving the utilization rate and economic benefits of the orchard. In this study, there was a significant accumulation of amino acids, sugars, and organic acids related to the unique taste of *Morchella* by intercropping cultivation. In addition, placing the density of six exogenous nutrient bags per square meter is perhaps the most suitable for yield formation. Adding pear sawdust to nutrient bags can significantly increase the yield of morel and decrease the relative abundance of soil-borne fungal pathogens while increasing beneficial soil bacteria abundance. This study provides novel insights into the establishment and improvement of the pear-*Morchella* cultivation model formation.

## Figures and Tables

**Table 1:** Information of differential amino acids, sugars, or acids in intercropping *M. sextelata*.

	Name	Fold Change	*p*-Value	VIP Value	Difference
Amino acids	Phenylalanine	1.7058	0.0343	1.0678	up
	Lysine	2.0794	0.0037	1.2108	up
	Proline	2.4310	0.0001	1.2260	up
	Citrulline	1.5402	0.0035	1.2059	up
	Ornithine	2.4677	0.0002	1.2256	up
	Tryptophan	0.4186	0.0072	1.1676	down
Sugars or acids	Arabinitol	2.3655	0.0031	1.1971	up
	Glucosamine	2.3790	0.0009	1.1899	up
	Quinic acid	2.0403	0.0128	1.1679	up
	Fumaric acid	3.9155	0.0002	1.1960	up
	Malic acid	1.9617	0.0028	1.1873	up
	Mannose	0.5011	0.0137	1.1158	down

## Data Availability

The original contributions presented in the study are included in the article. Further inquiries can be directed to the corresponding author.
